# The biomarkers related to immune related adverse events caused by immune checkpoint inhibitors

**DOI:** 10.1186/s13046-020-01749-x

**Published:** 2020-12-14

**Authors:** Xiao-Hui Jia, Lu-Ying Geng, Pan-Pan Jiang, Hong Xu, Ke-Jun Nan, Yu Yao, Li-Li Jiang, Hong Sun, Tian-Jie Qin, Hui Guo

**Affiliations:** 1 Department of Medical Oncology, The First Affiliated Hospital of Xi’an Jiaotong University, Xi’an, Shaanxi China; 2 Oncology Hospital, Xi’an International Medical Center Hospital, Xi’an, Shaanxi China; 3 Key Laboratory of Environment and Genes Related to Diseases, Xi’an Jiaotong University, Ministry of Education of China, Xi’an, Shaanxi China; 4 Translational Medicine Center, The First Affiliated Hospital of Xian Jiaotong University, Xi’an, Shaanxi China

## Abstract

The enthusiasm for immune checkpoint inhibitors (ICIs), an efficient tumor treatment model different from traditional treatment, is based on their unprecedented antitumor effect, but the occurrence of immune-related adverse events (irAEs) is an obstacle to the prospect of ICI treatment. IrAEs are a discrete toxicity caused by the nonspecific activation of the immune system and can affect almost all tissues and organs. Currently, research on biomarkers mainly focuses on the gastrointestinal tract, endocrine system, skin and lung. Several potential hypotheses concentrate on the overactivation of the immune system, excessive release of inflammatory cytokines, elevated levels of pre-existing autoantibodies, and presence of common antigens between tumors and normal tissues. This review lists the current biomarkers that might predict irAEs and their possible mechanisms for both nonspecific and organ-specific biomarkers. However, the prediction of irAEs remains a major clinical challenge to screen and identify patients who are susceptible to irAEs and likely to benefit from ICIs.

**Supplementary Information:**

**Supplementary information** accompanies this paper at 10.1186/s13046-020-01749-x.

## Background

In recent years, immune checkpoint inhibitors (ICIs) have achieved gratifying effects in a wide variety of tumors, including melanoma [1], renal cell carcinoma [2] and non-small cell lung cancer (NSCLC) [3], which greatly changed the traditional tumor treatment strategy and brought more survival benefits to patients [4, 5]. However, much of the enthusiasm for ICIs is based on long-term survival benefits, which occur in only a few patients. The survival benefit of patients is not only determined by the efficacy but also affected by adverse events. While ICIs represent a new field against cancer, they have also produced a unique set of immune-related adverse events (irAEs) that could have serious or even fatal consequences. Only by improving efficacy and reducing toxicity as much as possible could patient survival be improved.

Undeniably, irAEs are very common, depending on the ICI mechanism. The application of ICIs destroys the mechanism that might protect tissues from autoimmune response damage [6], enhances the activity of T cells against antigens presented in tumors and healthy tissues [7], and increases the level of pre-existing autoantibodies and inflammatory factors [7], leading to a series of irAEs. However, current research on the mechanisms of irAEs is still in the early stage, and there are no recognized and universal mechanisms to explain irAEs. Strikingly, discrete toxicities caused by the nonspecific activation of the immune system could affect almost all tissues and organs. Among them, irAEs of the digestive system, endocrine organs and lungs are more common, and the heart, liver, kidneys, nerves, and eyes are relatively less affected [8]. The major fatal toxicities are cardiotoxicity, neurotoxicity and interstitial pneumonia, which are as high as 45% [9]. In some studies, the reported incidence was as high as 90% for any grade irAEs from ICI monotherapy [10]. A meta-analysis indicated an overall incidence over 70% with anti-cytotoxic T-lymphocyte antigen-4 (anti-CTLA-4) monotherapy (ipilimumab, IPI) [11] and 27–78% in phase 3 trials of anti-programmed cell death protein 1 (anti-PD-1)/anti-programmed death ligand-1 (anti-PD-L1) agents [12–14]. Serious irAEs could lead to irreversible outcomes. The clinical characteristics of irAEs are relatively hidden with subtle imaging changes and are difficult to determine in the early stage. Therefore, some of the major clinical challenges include the early identification of patients who are susceptible to irAEs before they occur and the monitoring of the development of irAEs. Additionally, the common clinical strategy is mostly the combination of immunotherapy with chemotherapy or targeted therapy, so it is difficult to judge whether the adverse events are caused by immunotherapy alone, which suggests that it is important to accurately identify irAEs. Therefore, it is imperative to develop predictive markers for the occurrence of irAEs, to screen high-risk groups, to monitor the change in irAEs and to judge the outcome of irAEs to further optimize the benefit of patients and minimize the risk of toxicity.

Many factors, such as sex and tumor type, might be able to predict the occurrence of irAEs. From extensive literature reports and clinical experience, it was found that males had a better response than females, but females were more likely to suffer irAEs [15]. Moreover, it is known that females are more susceptible to autoimmune diseases [16], which might be related to some sex-specific factors. In addition, a meta-analysis suggested that different tumor types had different immune microenvironments that might drive tissue-specific irAEs [17]. For example, dermatitis and arthritis were more common in melanoma patients than in renal cell carcinoma patients, while pneumonia and dyspnea were less common in melanoma patients [17]. However, these factors cannot accurately predict the occurrence of irAEs, making it difficult to achieve the purpose of early screening and early detection. Moreover, most of the previous studies focused on identifying immunotherapy advantage populations, and biomarkers of irAEs could help identify patients susceptible to severe irAEs as exclusion populations for immunotherapy. Therefore, considering the unique clinical value of biomarkers, as well as their convenience and accuracy, we listed the currently known predictive biomarkers of irAEs in this review to help better understand irAEs and provide clinical clues for subsequent studies.

## Methods

Original articles on irAEs up to January 2020 were screened in Pubmed, Embase and Cochrane library. Medical Subject Headings are used to search for the terms carcinoma, immune checkpoint inhibitor and irAEs, including various adverse events. In this review, two investigators (JPP and XH) independently extracted data. Each study recorded the following information: biomarker, author, year, cancer type, patient number, treatment, correlation between biomarker and irAEs as well as possible hypothesis (Tables 1 and 2 for details). Each study was reviewed several times to ensure that no data was lost or mislabeled. If there are any objections, it was resolved through discussion or a third researcher to decide whether to include or not. For incomplete documents, try to contact the original author to supplement. The quality of included studies was assessed using the Cochrane Hand book 5.1.0 recommended risk of bias assessment tool. Including: (1) random allocation method; (2) allocation concealment; (3) whether to adopt a blind method for the participants and researchers; (4) whether the outcome was assessed by blind method; (5) completeness of outcome data; (6) Selective reporting of outcomes; (7) Other bias. The quality evaluation was conducted independently by two researchers. If there were different opinions, the decision was made through discussion or by referring to the viewpoint of the third researcher.

**Table 1:** Nonspecific biomarker of irAEs

Biomarker	Author	Year	Cancer type	Patient number	Treatment	Correlation between biomarker and irAEs	Possible hypothesis
CRP	Abolhassani AR [18]	2019	MM	37	Anti-PD-1 Anti-CTLA-4	CRP elevation can predict the onset of irAEs in patients treated with ICIs in the absence of infectious disease.	Tumor-promoting inflammation could cause a systemic inflammatory response;CRP level was positively associated with the infiltration of CD8 + T cell and Treg cell which could activate the systemic inflammatory response.
IL-6	Okiyama N [19]	2017	MM	22	Anti-PD-1	The IL-6 level was significantly increased in the patients with psoriasiform dermatitis after nivolumab treatment.	Overactivation of the immune system;Excessive release of inflammatory cytokines.
	Valpione S [15]	2018	MM	140	Anti-CTLA 4	A lower baseline level of IL-6 was strongly associated with the development of irAEs.	
Blood cell count	Fujisawa Y [20]	2017	MM	101	Anti-PD-1	The increase of WBC counts and the decrease of relative lymphocyte counts were closely related to the incidence of grade 3–4 irAEs.	Conventional blood cell counts could be a crude reflection of the body’s immune state, but the mechanism is unclear.
	Diehl A [21]	2017	Multiple solid tumors (lung cancer, MM, RCC, urothelial, HNSCC, Merkel cell carcinoma, colon cancer)	167	Anti-PD-1	Higher baseline and increase of absolute lymphocyte and eosinophil counts after ICIs treatment were strongly associated with the development of irAEs.	
	Nakamura Y [22]	2019	MM	45	Anti-PD-1	The elevation of absolute eosinophil count at baseline and relative eosinophil count at 1 month might be valuable biomarkers to predicte endocrine irAEs.	
Cytokines	Khan S [23]	2019	Multiple solid tumors (lung cancer, kidney cancer, MM, head/neck cancer, liver cancer, bladder cancer)	65	Anti-PD-1/L1 Anti-CTLA 4	The up-regulation of various cytokines after ICIs treatment was closely related to the occurrence of irAEs, especially the induced CXCL9, 10, 11 and 13.	Activate T cell;Excessive release of cytokines;Various cytokines have powerful pro-inflammatory activities, including stimulating immune cell recruitment, proliferation, survival, differentiation, and effector functions, and many of these cytokines (such as IL-1A, IL-1B, IL-2, IFN 2, and IL-12P70) are associated with inflammation, which is the basis of autoimmune diseases.
	Lim SY [24]	2019	MM	98	Anti-PD-1 Anti-CTLA 4	Eleven cytokines, including G-CSF, GMCSF, Fractalkine, FGF-2, IFN-2, IL-12p70, IL-1a, IL-3 1B, IL-1RA, IL-2, IL-13, were significantly upregulated in patients with severe irAEs at baseline and early during treatment.	
TMB	Bomze D [25]	2019	Multiple solid tumors	16,397	Anti-PD-1	There is a significant positive correlation between high TMB and irAEs during anti-PD-1 therapy in a variety of solid tumors	While fighting against neoantigens, T cells could also cross-react with the corresponding wild-type antigens in normal tissues, resulting in damage to normal tissues.
sCLTA-4	Pistillo MP [26]	2018	MM	113	Anti-CTLA-4	Higher baseline levels of sCTLA-4 were closely associated with irAEs, especially the gastrointestinal adverse events.	Elevated levels of sCTLA-4 might block the interactions between full-length CTLA-4 expressed by autoreactive T cells and Tregs as well as B7 ligands, thus enhance the cytotoxicity of T cells and reduce the immunosuppression function of Treg cell.

*irAEs* immune related adverse events, *ICIs* immune checkpoint inhibitors, *CRP* C reactive protein, *MM* malignant melanoma, *Anti-PD-1/L1* anti-programmed cell death protein 1/ligand 1, *Anti-CTLA-4* anti-cytotoxic T lymphocyte associated antigen-4, *IL-6* interleukin 6, *RCC* renal cell carcinoma, *HNSCC* head and neck squamous cell carcinoma, *WBC* white blood cell, *NLR* neutrophil-lymphocyte ratio, *TMB* tumor mutation burden, *sCLTA-4* soluble CTLA-4, *flCTLA-4* full-length CTLA-4

**Table 2:** Organ-specific biomarker of irAEs

irAEs		Biomarker	Author	Year	Cancer type	Patient number	Treatment	Correlation between biomarker and irAEs	Possible hypothesis
GI		The disorder of gut microbiome	Chaput N [27]	2017	MM	26	Anti-CTLA-4	Baseline stool samples without bacteroidetes and with high levels of firmicutes were more likely to develop immune-related colitis.	Impaired metabolism of beneficial bacteria;Decreased beneficial bacteria that inhibit inflammation;Microbial-derived products trigger an innate immune response.
		CD177 and CEACAM1	Shahabi V [28]	2013	MM	162	Anti-CTLA-4	CD177 and CEACAM1 were highly expressed at baseline and post-baseline in patients with GI irAEs.	CD177 and CEACAM1, as activation markers of neutrophils, are involved in immune-mediated intestinal disease.
		Peripheral blood mRNA expression (CCL3, CCR3, IL-5, IL-8 and PTGS2)	Friedlander P [8]	2018	MM	210	Anti-CTLA-4	Peripheral blood gene expression characteristics (mainly CCL3, CCR3, IL-5, IL-8 and PTGS2) were closely related to the immune-related diarrhea, especially grade 2–4 diarrhea.	Up-regulated genes such as CCL3, CCR3, IL-5, IL-8 and PTGS2 are involved in inflammatory immune response.
		IL-17	Tarhini AA [29]	2015	MM	35	Anti-CTLA-4	Upregulation of IL-17 level at baseline and 6 weeks after ICIs treatment showed a noteworthy correlation with grade 3 diarrhea/colitis.	IL-17, one of the up-regulated central inflammatory cytokines in IBD, was usually inhibited by CTLA-4, but the intervention of ICIs disrupted this ecological balance.
		HLA allele	Hasan Ali O [30]	2019	NSCLC, MM	102	Anti-PD-1 Anti-CTLA-4	There was a significant correlation between HLA type II variant HLA-dqb1 * 03:01 and immune-related colitis.	The presence of common antigens between the tumor and colon tissue causes misleading damage to the gastrointestinal tract by the immune system.
Immune-related pneumonia		CD74	Tahir SA [31]	2019	Bladder cancer, prostate cancer	8	Anti-CTLA-4 + Anti-PD-1	The increase of autoantibody CD74 level after ICIs treatment was notably correlated with immune-related pneumonia	CD74 stimulates the release of inflammatory mediators;There is a common antigen between the tumor and lung; ICIs disrupts the mechanism that inhibits the inflammatory response of Th2 cells.
Endocrine disorder		Preexisting abnormal antibodies	Toi Y [32]	2019	NSCLC	137	Anti-PD-1	Preexisting abnormal antibodies was independently associated with irAEs. Patients with positive RF are more likely to develop dermal irAEs, and thyroid dysfunction is more common in patients with positive antithyroid antibody.	T-cells enhance the effect of PD-1 antibody, and might in turn induce B-cells to produce autoantibodies, which will lead to the toxic accumulation effect of pre-existing abnormal autoantibodies, and finally trigger irAEs.
	Thyroid dysfunction	Abnormal TPOAb	Gay S [33]	2019	NSCLC, MPM	28	Anti-CTLA-4 + Anti-PD-1	There was a association between widespread thyroid hypoechogenicity, decreased thyroid volume, elevated TPOAb after ICIs treatment and thyroid dysfunction.	ICIs enhance T cell activity against antigens present in healthy tissues and increase pre-existing autoantibody levels.
	Hypophysitis	GNAL and ITM2B	Tahir SA [31]	2019	Prostate cancer, MM, RCC	9	ICI therapy	Elevated levels of autoantibodies GNAL and ITM2B are closely related to the immune-related hypophysitis.	The qualitative difference in the autoreactive effector T cells between anti-PD-1 / PD-L1 and anti-CTLA-4 treatment; The pituitary endocrine cells themselves might express CTLA-4, making hypophysis a direct target for anti-CTLA-4 antibodies.
Dermatologic toxicity		HLA alleles	Hasan Ali O [30]	2019	NSCLC, MM	102	Anti-PD-1 Anti-CTLA-4	HLA- drb1 *11:01 was observably related with itching.	The presence of common antigens between the tumor and skin causes dermatologic misleading damage.
		IL-17	Johnson D [34]	2019	MM	3	Anti-PD-1	Psoriasiform dermatologic toxicity induced by PD-1 inhibitor subsided after treatment with systemic interleukin-IL17A blockade.	ICIs enhance the Th17-mediated immune response in susceptible patients.

*irAEs* immune related adverse events, *GI* gastrointestinal irAEs, *MM* malignant melanoma, *Anti-CTLA-4* anti-cytotoxic T lymphocyte associated antigen-4, *Anti-PD-1/L1* anti-programmed cell death protein 1/ligand 1, *IL-8* interleukin 8, *IBD* inflammatory bowel disease, *HLA* human leukocyte antigen, *IL-17* interleukin 17, *NSCLC* non-small cell lung cancer, *MPM* malignant pleural mesothelioma, *RCC* renal cell carcinoma, *ICI* immune checkpoint inhibitor

## Potential biomarkers associated with irAEs

IrAEs could be found in all organs, and its forms are also varied between target organs and ICIs. Based on the current data, several potential biomarkers have been identified to predict irAEs, as described below.

### Nonspecific biomarkers

**Fig. 1:**
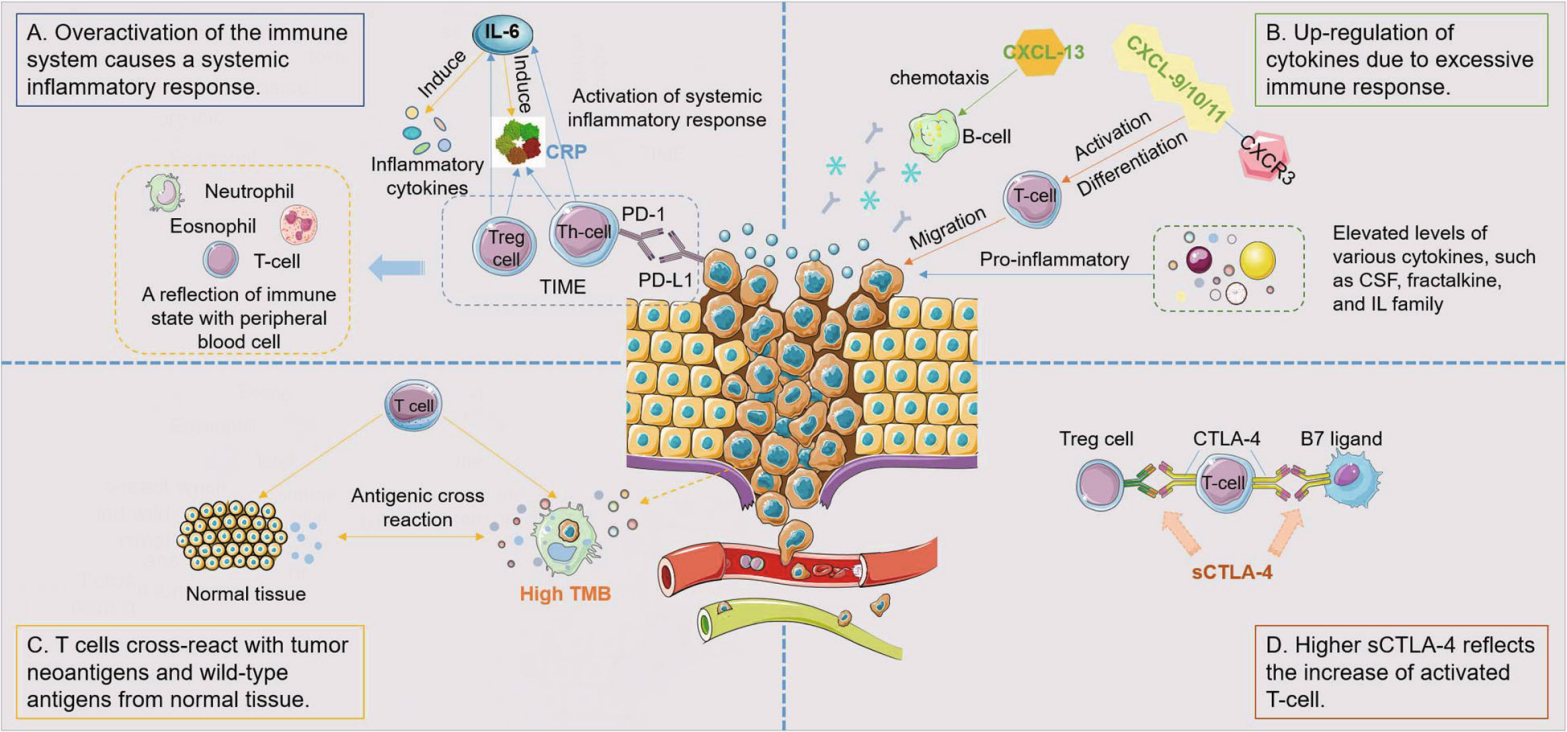
Possible mechanisms of nonspecific biomarkers of irAEs. **a** Possible mechanisms of CRP, IL-6 and blood cell count. Tumor-promoting inflammation can cause overactivation of the immune system and trigger a systemic inflammatory response, leading to some non-specific irAEs. **b** Possible mechanisms of cytokines. CXCL9/10/11, involved in the establishment of CXCL9/10/11-CXCR3 axis in tumor microenvironment, is chemokine to activate T cells and regulate the differentiation, activation and migration of immune cells. CXCL 13 is the B-cell chemoattractant. Their upregulation is associated with a variety of autoimmune diseases, which is considered to be a key cytokine axis closely related to irAEs. And the increased expression of 11 cytokines represented by CSF, fractalkine, and IL family is closely related to severe irAEs. **c** Possible mechanisms of TMB. Dead tumor cells can released neoantigens which produce a high TMB. While fighting against neoantigens, T cells could also cross-react with the corresponding wild-type antigens in normal tissues, resulting in damage to normal tissues. **d** Possible mechanisms of sCTLA-4. Elevated levels of sCTLA-4 might block the interactions between full-length CTLA-4 expressed by autoreactive T cells and Tregs as well as B7 ligands, thus enhance the cytotoxicity of T cells and reduce the immunosuppression function of Treg cell. PD-1/L1, programmed cell death protein 1/ligand 1; TIME, tumor immune microenvironment; IL-6, interleukin 6; CRP, C reactive protein; Th cell, helper T cell; CSF, colony stimulating factor; TMB, tumor mutation burden; CTLA-4, cytotoxic T lymphocyte associated antigen-4; sCTLA-4, soluble cytotoxic T lymphocyte associated antigen-4

In cases of nonspecific symptoms such as fever, cough, and fatigue, it is often difficult to make timely and rapid clinical decisions due to complex screening imaging diagnosis or hematological examination because these are time consuming and require cautious differential diagnosis. Therefore, there is an urgent need to develop easily detectable biomarkers to identify nonspecific irAEs. The known nonspecific biomarkers of irAEs are shown in Table 1, and the possible mechanisms are shown in Fig. 1. Additionally, the quality assessment of the included studies is shown in supplementary Figure 1.

#### C-reactive protein

The theory that high C-reactive protein (CRP) levels are closely associated with poor prognosis is well known [35], and the current research focus has gradually shifted to the relationship between CRP levels and irAEs. A retrospective study found that CRP was increased in patients with irAEs, such as pituitary inflammation, hepatitis, thyroiditis and autoimmune colitis (upper limit of normal (ULN) 5 mg/L), but elevated CRP levels did not correlate with the severity of organ damage [18]. However, this hypothesis still needs to be verified by a larger patient cohort. Currently, the mechanism of the correlation between CRP and irAEs is unclear. CRP, as a representative acute phase reactant, is part of the nonspecific immune mechanism that reflects the presence of systemic inflammation in the host [18] and represents a high level of immunogenicity and tumor burden. Inflammatory cells could be attracted to sites of neoplasia and may promote cancer cells [36–38] which is a typical example of tumor cells conscripting normal cells to enhan ce tumor growth potential. This tumor-promoting inflammation is an important means to acquire the necessary capabilities for tumor cells [39], which is considered to indicate poor prognosis in many tumors [40, 41]. Additionally, some studies have shown that the elevation in CRP baseline levels was positively associated with the infiltration of CD8+ T cells and regulatory T (Treg) cells [41], and highly activated effector T cells could activate the systemic inflammatory response, which might be associated with nonspecific irAEs [42] (Fig. 1a).

CRP as a biomarker has the advantages of easy sample collection, simple measurement and convenient analysis. However, the CRP level is susceptible to a variety of physiological and pathological factors, such as acute or chronic infections, anti-infective and anti-inflammatory drugs and autoimmune diseases, which suggests that a single estimation of the CRP level is not able to accurately and objectively predict irAEs. Therefore, in short, patients were considered to suffer from irAEs if their CRP increased at least twice during the two-week interval, the procalcitonin level was low, and there was no evidence of infection (negative for etiological culture and serology) [18].

#### Interleukin − 6

Similar to the association with poor ICI efficacy [42, 43], an important observation was that a high interleukin-6 (IL-6) level after ICI treatment was closely related to irAEs, such as Crohn’s disease [44] and psoriasiform dermatitis [19]. In contrast, a previous study of malignant melanoma patients using a CTLA-4 inhibitor found that a lower baseline level of IL-6 was strongly associated with the development of irAEs [15], including colitis [27]. These results indicated that both low baseline IL-6 levels and increased IL-6 levels after ICI treatment might predict the occurrence of irAEs. This might be explained by the important role of IL-6 in inflammation. IL-6 has a two-sided effect on the development of tumors, both preventing tumor formation and promoting tumor progression [42, 45]. IL-6 plays an active role in innate and adaptive immunity, such as the activation of helper T (Th) cells, inhibition of Treg cells, and differentiation of B cells [46], and these overactive manifestations of the immune system are closely related to irAEs [7]. Another important deduction is the excessive release of inflammatory cytokines [7]. IL-6 plays an important role in the tumor-related systemic inflammatory response, not only as a major inducer of CRP [47] but also as a direct inducer of anti-inflammatory molecules [48], which are also considered potential mechanisms by which IL-6 participates in the occurrence of irAEs. The overall hypothesis is shown in Fig. 1a. However, so far, there is no consensus.

Meanwhile, recent studies have shown that the CRP level in peripheral blood might act as a surrogate for IL-6 activity in vivo based on cytokine release syndrome (CRS) after treatment with chimeric antigen receptor T-cells (CAR-T cells) in leukemia [49]. A significant proportion of patients with irAEs had characteristics similar to those of patients with CRS [6], and IL-6 was significantly increased in CRS [49, 50], which directly demonstrated the effectiveness of IL-6 as an irAE biomarker.

#### Blood cell count

Blood counts have received widespread attention as a highly available specimen and a signal of irAEs. Fujisawa Y et al., by analyzing the fluctuation of blood counts on or before the day of irAEs in melanoma patients treated with nivolumab, found that the increase in white blood cell counts and the decrease in relative lymphocyte counts were closely related to the incidence of grade 3–4 irAEs, especially pulmonary irAEs [20]. A previous analysis suggested that a higher baseline and increase in absolute lymphocyte counts (ALC) and absolute eosinophil counts (AEC) after ICI treatment were strongly associated with the development of irAEs in patients with solid tumors (including lung cancer, kidney cancer, melanoma, etc.) treated with anti-PD-1 antibodies (baseline ALC > 2000, AEC ≥ 100) [21]. Encouragingly, studies by Nakamura Y et al. in melanoma patients treated with anti-PD-1 antibody showed that AEC > 240/μL at baseline and relative eosinophil count at 1 month > 3.2% could be valuable biomarkers for predicting endocrine irAEs [22]. Conversely, a study by Leila Khoja et al. revealed that in melanoma patients treated with IPI, the neutrophil-lymphocyte ratio (NLR), platelet-lymphocyte ratio (PLR) and eosinophil-lymphocyte ratio (ELR) were not associated with toxicity but only with the response to ICI treatment [51]. The reason for this paradox might be the difference in pathological changes caused by different types of ICIs.

Conventional blood cell counts are known to be a crude reflection of the body’s immune state and are crucial in classic cellular, humoral, and tumor immunity (Fig. 1a). However, considering the involvement of different types of ICIs and various affected sites, the mechanism is complex and diverse, and no unified conclusion has been reached yet. Moreover, the blood cell count has certain limitations as a biomarker. Similar to CRP, it is primarily affected by the patient’s inflammatory state, physical condition, recent new infections or other diseases unrelated to the original tumor and to a large extent by myelosuppression due to chemotherapy or radiotherapy. Therefore, the accuracy and reliability of blood cell count as a predictor of irAEs still need to be further weighed and verified.

#### Cytokines

Cytokines, a kind of small-molecule protein with extensive biological activities, play a crucial role in a variety of life activities, such as innate and adaptive immunity, tumor growth, and the inflammatory response [52]. Among the 40 cytokines/chemokines assessed in plasma, Shaheen Khan et al. found that compared with the baseline level before ICI treatment, the upregulation of various cytokines after ICI treatment, especially induced CXCL9, 10, 11 and 13, was closely related to the occurrence of irAEs [23]. CXCL9/10/11, involved in the establishment of the CXCL9/10/11-CXCR3 axis in the tumor microenvironment, is a chemokine that activates T cells [23], which could regulate the differentiation, activation and migration of immune cells and effectively inhibit tumor growth [53] (Fig. 1b). Moreover, previous studies indicated that they were associated with a variety of autoimmune diseases, including thyroiditis, inflammatory bowel disease (IBD), and systemic sclerosis [52, 54]. Therefore, it is considered to be a key cytokine axis closely related to irAEs, but as yet, it has not been observed to be associated with organ-specific irAEs. CXCL 13, as a B-cell chemoattractant, is mainly expressed in mature B-cells, CD4+ follicular Th cells and activated Treg cells [54, 55] and participates in a series of autoimmune and inflammatory diseases, including multiple sclerosis, rheumatoid arthritis and systemic lupus erythematosus [56–59] (Fig. 1b). At the same time, the levels of IL-6, CXCL2, CCL20, CXCL8 and CCL23 in patients with irAEs were also significantly higher than in those without irAEs [23]. Therefore, this might support the hypothesis that ICI treatment induced an excessive immune storm that ultimately led to irAEs. Furthermore, in another retrospective study, the elevation of cytokines was found prior to ICI treatment [60], which might be in favor of the assumption that ICI treatment triggered a silent subclinical inflammatory response. Remarkably, the complex tumor microenvironment is a critical regulator involved in immune escape, progression, and distant metastasis [61]. High expression of hepatocyte growth factor and colony-stimulating factor-1 (CSF-1) has also been found to promote immune tolerance in the immunosuppressive microenvironment of hepatocellular carcinoma as an inflammation-associated tumor [62]. Interestingly, the study of Lim SY et al. also confirmed that the increased expression of 11 cytokines represented by the CSF, fractalkine, and IL families is closely related to severe irAEs [24]. These cytokines not only have strong pro-inflammatory activity but also participate in the inflammation of various autoimmune diseases [63, 64], so they could predict irAEs to a certain extent. In summary, cytokines are a potential biomarker for predicting irAEs, but no organ specificity has been found.

#### Tumor mutation burden

Tumor mutation burden (TMB) has been widely promoted as a useful biomarker for predicting the expected therapeutic response of tumors treated with immunotherapy. Then, a challenging question arises: does TMB have the same predictive power for irAEs? A large retrospective analysis of 16,411,749 irAE reports from 5,160,064 patients by David Bomze et al. revealed a significant positive correlation between high TMB and irAEs during anti-PD-1 therapy in a variety of solid tumors [25]. One contributing mechanism is the antigen spreading theory (Fig. 1c). After ICIs are taken, tumor cells die and release antigens, including neoantigens, which results in high TMB [25]. While fighting against neoantigens, T cells could also cross-react with the corresponding wild-type antigens in normal tissues [65], resulting in damage to normal tissues. The association between high TMB and irAEs was via the release of potential neoantigens, which is consistent with the theory of a positive correlation between high TMB and improved response to anti-PD-1 therapy. Regrettably, however, no studies have shown a link between TMB and organ-specific irAEs or their severity. There is currently no evidence linking TMB to irAEs caused by other ICIs, such as PD-L1 and CTLA-4 inhibitors. These valuable clinical questions are expected to be addressed by further research. In conclusion, high TMB might be a potential biomarker for assessing the risk of irAEs in vulnerable patient groups, but it needs to be validated and further explored in large prospective clinical studies.

#### sCLTA-4 level

Maria Pia Pistillo et al. suggested that higher baseline levels of soluble CTLA-4 (sCTLA-4 > 200 pg/ml) were closely associated with irAEs in melanoma patients treated with IPI, especially gastrointestinal irAEs [26]. One possible hypothesis is based on the dysregulation of autoreactive T cells (Fig. 1d). IPI exerts a powerful antitumor effect by blocking CTLA-4 binding to CD80 and CD86 ligands on antigen-presenting cells, which leads to T cell activation, intracellular congestion, and tumor-specific effects [66, 67]. Maria Pia Pistillo and colleagues suggested that elevated sCTLA-4 levels might block the interactions between full-length CTLA-4 expressed by autoreactive T cells and Tregs as well as B7 ligands, thus enhancing the cytotoxicity of T cells and reducing the immunosuppressive function of Treg cells [26]. In other words, a high level of sCTLA-4 reflects the increase in T cell activation after IPI treatment. In addition, as some previous studies have shown, an increase in sCTLA-4 levels was associated with longer overall survival (OS) in a variety of tumors, such as malignant melanoma [26], malignant mesothelioma [68], and NSCLC [69]. Moreover, many studies pointed out that the occurrence of irAEs was positively correlated with a better efficacy of ICIs [42, 70], which also supported that sCTLA-4 might be a promising biomarker for predicting irAEs. However, sCTLA-4 is mainly produced by Treg cells, which are mainly involved in immune escape, and some tumor cells can also produce and release sCTLA-4 into the blood [71]. Therefore, to some extent, a high level of sCTLA-4 could also be used as a marker of tumor immune escape and high tumor burden, which is contradictory to the above theory. Overall, the specific mechanism of sCTLA-4 as a biomarker for predicting irAEs and the association between sCTLA-4 levels and irAE grades still need further exploration.

### Organ-specific biomarkers

**Fig. 2:**
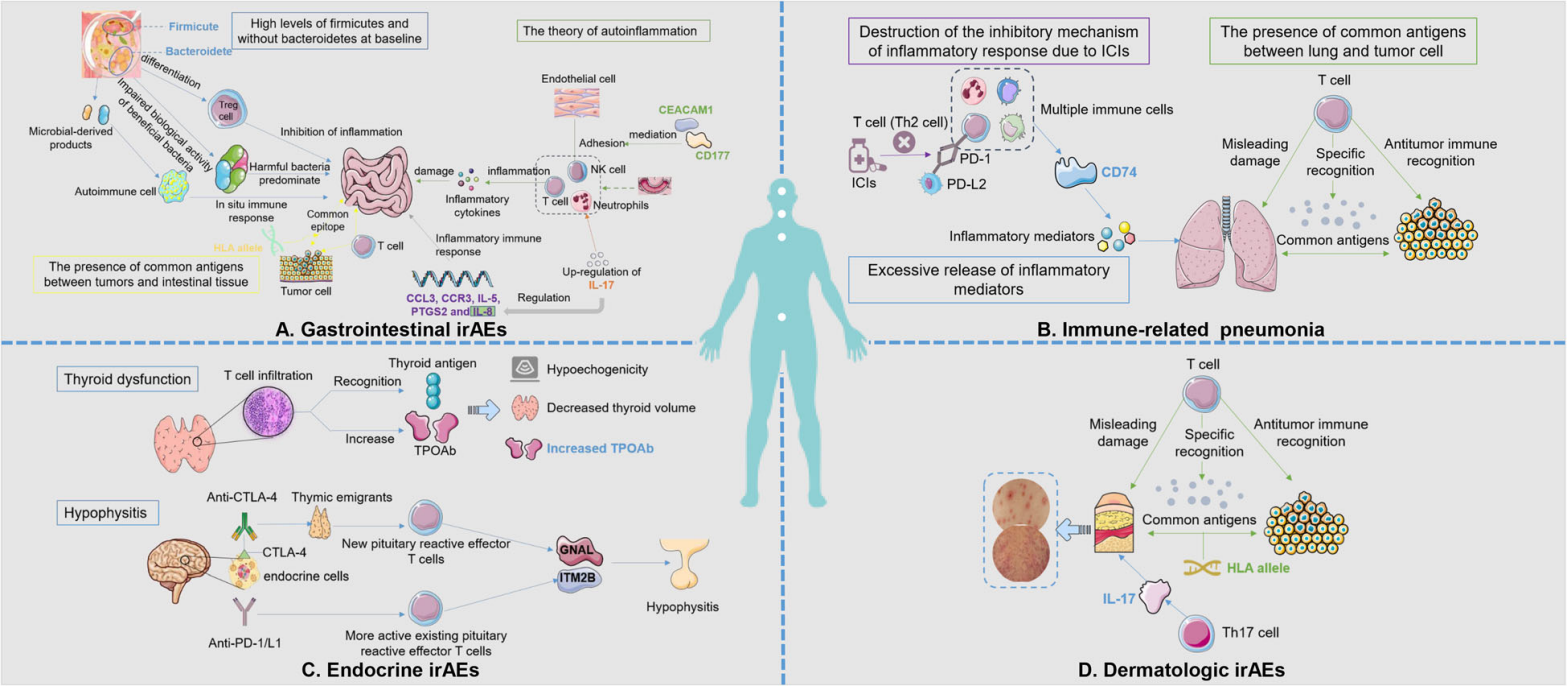
Possible mechanisms of organ-specific biomarkers of irAEs. **a** Possible mechanisms of biomarkers associated with gastrointestinal irAEs. The main biomarkers for predicting gastrointestinal irAEs are disordered gut microbiome, CD177 and CEACAM1, peripheral blood mRNA expression (CCL3, CCR3, IL-5, IL-8 and PTGS2), IL-17 and HLA allele. a) One possible mechanism for disordered gut microbiome as a biomarker is that ICIs disrupt the stable microbial system in the gut, resulting in impaired metabolism of beneficial bacteria and a decrease in beneficial bacteria that inhibit inflammation. Another proposed hypothesis is that the microbial-derived products of intestinal disorders could trigger an in situ immune response that might eventually lead to the activation of self-reactive immune cells, causing local intestinal tissue damage. b) CD177 and CEACAM1 are involved in the activation of neutrophils and are thus closely related to immune-mediated intestinal diseases. c) Up-regulated genes such as CCL3, CCR3, IL-5, IL-8 and PTGS2 are involved in inflammatory immune response. d) IL-17, one of the up-regulated central inflammatory cytokines in IBD, was usually inhibited by CTLA-4, but the intervention of ICIs disrupted this ecological balance. e) HLA gene acts an important role in antigen expression and immune tolerance. The presence of common antigens between the tumor and colon tissue causes misleading damage to the gastrointestinal tract by the immune system. **b** Possible mechanisms of immune-related pneumonia. One possible hypothesis is that CD74 stimulates the release of inflammatory mediators. Additionally, there is a common antigen between the tumor and lung, causing misleading damage to the lung by the immune system. And another new hypothesis is ICIs disrupts the mechanism that inhibits the inflammatory response of Th2 cells. **c** Possible mechanisms of endocrine irAEs. a) Abnormal TPOAb might predict the thyroid dysfunction because ICIs enhance T cell activity against antigens present in healthy tissues and increase pre-existing autoantibody levels. b) Elevated levels of autoantibodies GNAL and ITM2B are closely related to the immune-related hypophysitis. Possible mechanism is anti-CTLA-4 could generate new reactive effector T cells in the pituitary, while anti-PD-1 / PD-L1 is more likely to make existing pituitary reactive effector T cells more active. Another hypothesis is that the pituitary endocrine cells themselves might express CTLA-4, making hypophysis a direct target for anti-CTLA-4 antibodies and causing hypophysis destruction. **d** Possible mechanisms of dermatologic toxicity. a) HLA gene mediates a variety of autoimmune diseases and the presence of common antigens between the tumor and skin causes dermatologic misleading damage. b) Th17 cells could induce the transcription of IL-17 cytokines. ICIs enhance the Th17-mediated immune response in susceptible patients, resulting in increased IL-17 levels and ultimately inducing dermatologic irAEs. IL-17, interleukin 17; NK cell, natural killer cell; ICIs, immune checkpoint inhibitors; Th cell, helper T cell; PD-1/L1/L2, programmed cell death protein 1/ligand 1/ligand 2; CTLA-4, cytotoxic T lymphocyte associated antigen-4; HLA, human leukocyte antigen

Not all patients taking ICI treatment will suffer the same irAEs. Real world results have shown a variety of complex conditions, such as organ specificity and drug specificity, which might be influenced by the individual differences of patients, disease characteristics, drug types, regional culture and other mixed factors. Organ-specific irAEs are mainly a discrete toxicity caused by the nonspecific activation of the immune system, which could invade many organs, such as the gastrointestinal tract, endocrine system, and lungs. Different organs have different responses and affinities to ICI treatment. For example, colitis mostly occurs in patients taking IPI, while pneumonitis mostly occurs in patients taking anti-PD-1/PD-L1 [9]. What are the mechanisms of organ-specific irAEs? To date, no consensus has been reached. The hypotheses focus on the excessive release of inflammatory cytokines, overactivation of the immune system, amplification of preexisting abnormal antibodies, and misleading damage caused by the presence of common antigens between tumors and normal tissues. The organ-specific biomarkers of irAEs are shown in Table 2, and the possible mechanisms are shown in Fig. 2. The quality assessment of the included studies is shown in supplementary Figure 2.

#### Gastrointestinal irAEs

Gastrointestinal (GI) irAEs, mainly manifested as diarrhea, colitis, and IBD, are the most common irAEs in ICI treatment [4, 72], especially IPI [73–75], and most occur in 2–3 cycles after ICI treatment [76]. In most cases, GI irAEs were tolerable and did not result in the termination of ICI treatment. However, when grade 2–4 diarrhea occurs, effective treatment measures should be taken promptly, and immunotherapy should be terminated if necessary [77]. The main mechanism is based on the hypothesis that ICI triggers the activation of T cells, which in turn leads to an excessive increase in a range of inflammatory cytokines. Currently, biomarkers for predicting GI irAEs are the focus of research. The main biomarkers are as follows, and the possible mechanisms are shown in Fig. 2a.

##### The disorder of the gut microbiome

It is well known that colitis is closely related to the decrease in the biodiversity of the intestinal microbiome [27] and the destruction of the host-bacterial ecosystem [78]. A clinical study by high-throughput sequencing of fecal samples from patients with advanced malignant melanoma treated with IPI showed that a significant reduction in the percentage of Firmicutes after immunotherapy, at least 2 times lower than baseline, was associated with immune-related colitis [27]. Intriguingly, the microbiota of patients prone to colitis was rich in Firmicutes at baseline but significantly decreased after ICI treatment, while a higher proportion of Bacteroidetes was present in patients without colitis. However, another study claimed that Bacteroidetes were underrepresented in metastatic melanoma patients with immune-related colitis treated with IPI [79]. Therefore, in short, patients with baseline stool samples without Bacteroidetes and with high levels of Firmicutes were more likely to develop immune-related colitis. A stable microbial system in the gut is essential for maintaining the intestinal epithelial barrier and anti-inflammatory effects [80], and its disruption leads to impairments in the bacterial polyamine transport system and biosynthesis of thiamine, riboflavin and pantothenate [79], which might be a potential hypothesis. Bacteroidetes can inhibit inflammation by stimulating Treg cell differentiation [81, 82], so patients lacking Bacteroidetes are more likely to experience a local intestinal inflammatory response. Another proposed mechanism is that the microbial-derived products of intestinal disorders could trigger an in situ immune response that might eventually lead to the activation of self-reactive immune cells [83], causing local intestinal tissue damage.

##### CD177 and CEACAM1

Vafa Shahabi et al. analyzed the gene expression profiles of whole blood samples of melanoma patients treated with IPI and found that the neutrophil activation markers CD177 and CEACAM1 were highly expressed at baseline and postbaseline in patients with GI irAEs [28]. Moreover, the increase in CD177 occurred prior to the absolute neutrophil count increase in peripheral blood [28], indicating that CD177 might be a more sensitive early biomarker. The nonnegligible role of neutrophils in maintaining intestinal balance might support a reasonable hypothesis. CEACAM1 mediates activated neutrophils and other immune cells to adhere to endothelial cells [84, 85] involved in the pathogenesis of immune-mediated intestinal diseases. This could be verified in the colon biopsies of patients with GI irAEs, which revealed focal neutrophilic crypts and neutrophil infiltration in the lamina propria of the affected tissue [28, 86], the most prominent features of active colitis [28, 86]. This pathological change conforms to the theory of autoinflammation; that is, the local tissue inflammation of the host at the anatomical site leads to injury of the target organ [87]. In brief, increases in CD177 and CEACAM1 might be a signal for the occurrence of immune-related colitis.

##### Peripheral blood mRNA expression (CCL3, CCR3, IL-5, IL-8 and PTGS2)

A retrospective study based on two large clinical trials suggested that peripheral blood gene expression, mainly CCL3, CCR3, IL-5, IL-8 and PTGS2, which are involved in the inflammatory immune response, was closely related to immune-related diarrhea, especially grade 2–4 diarrhea [8]. In addition, previous studies have linked IL-8, CCR3, and CCL3 to diarrhea cases [88–90]. Furthermore, IL-8 expression is regulated by IL-17 [91], a cytokine clearly associated with the development of severe diarrhea/colitis [29]. This further increased the credibility of IL-8 as an irAE biomarker.

##### IL-17 and human leukocyte antigen allele

Ahmad A. Tarhini et al. reported that upregulation of IL-17 levels at baseline and 6 weeks after ICI treatment showed a noteworthy correlation with grade 3 diarrhea/colitis in melanoma patients treated with IPI [29]. IL-17 is a cytokine with a variety of inflammatory effects including the aggregation of neutrophils [92] and is one of the upregulated central inflammatory cytokines in IBD [93]. The increase in circulating IL-17 levels, which are usually inhibited by CTLA-4, might be a reflection of patients with subclinical colitis, but ICI intervention disrupts this ecological balance [92]. The inflammatory immune response might be the theoretical basis supporting IL-17 as a biomarker. In general, IL-17 might be developed as a promising biomarker to predict immune-related colitis due to its simple sample acquisition and easy detection.

Additionally, Omar Hasan Ali et al. identified a significant correlation between the human leukocyte antigen (HLA) type II variant HLA-dqb1 * 03:01 and immune-related colitis through the analysis of HLA allele typing [30]. The HLA gene plays a central role in antigen expression and immune tolerance [83]. Currently, the possible mechanism lies in the presence of common antigens between tumors and colon tissues, which enables colon tissues to be recognized by antigen-specific T cells, thus causing colon damage [30].

#### Immune-related pneumonia

Immune-related pneumonia is a change similar to interstitial pneumonia that is severe enough to be life-threatening [94]. Its clinical manifestations are complex and varied, and it is difficult to diagnose early and to distinguish from other pneumonia on imaging. Salahaldin A. Tahir et al. reported that the increase in an autoantibody against CD74 after ICI treatment was notably correlated with immune-related pneumonia, which suggested that CD74 plays an important role [31]. CD74, an autoantibody active protein, is an intracellular chaperone of MHC-II but is expressed on the cell membrane of immune cells, including macrophages [31] and could stimulate the release of inflammatory mediators [95]. Moreover, there was evidence that the presence of common antigens between tumor cells and lung tissues could enhance the recognition of lung antigens by specific T-cells [96], thus causing misleading damage to the lungs by the immune system. Alternatively, one new hypothesis was proposed that ICI disrupts the mechanism that inhibits the inflammatory response of Th2 cells in the body. Th2 cells participate in the development of pulmonary interstitial disease and the formation of eosinophils, and under normal circumstances, PD-1 interacts with PD-L2 to suppress the inflammatory response of Th2 cells, while ICI breaks this inhibition, thus producing immune-related pneumonia [97–101]. However, according to a study in children with severe viral pneumonia, CD74+ cells predominate in histopathological types with interstitial pneumonia as the pathological type [102], but no changes in CD74 have been observed in adults who developed pneumonia without immunotherapy. Therefore, it is reasonable to suspect that the change in CD74 level is specific to immune-associated pneumonia. In brief, the excessive release of inflammatory mediators, misleading damage due to the presence of common antigens between lung and tumor cells, and destruction of the inhibitory mechanism of the inflammatory response might be the causes of immune-related pneumonia (Fig. 2b).

#### Endocrine disorder

##### Preexisting abnormal antibodies

Multivariate analysis was conducted on patients with irAEs after ICI treatment, and the presence of preexisting abnormal antibodies, such as rheumatoid factor (RF), antinuclear antibody, antithyroglobulin, and antithyroid peroxidase, was independently correlated with irAEs [32]. Patients with positive RF (RF > 15 IU/mL at pretreatment) were more likely to develop dermal adverse events, and thyroid dysfunction was more common in patients with positive antithyroid antibody, either antithyroglobulin or antithyroid peroxidase at pretreatment [32]. Interestingly, in patients with high PD-L1 expression, the frequency of any existing antibodies and rheumatoid factors was slightly higher, but no significant correlation was observed between antibody expression levels and the severity of irAEs [32]. Some evidence illustrated that patients with abnormal autoantibodies before treatment had a greater chance of producing antibodies [32]. PD-1 is highly expressed in activated B-cells, and B cells are also regulated by T-cell-independent and -dependent mechanisms [103, 104]. T-cells enhance the effect of PD-1 antibody and might in turn induce B-cells to produce autoantibodies, which will lead to the toxic accumulation effect of pre-existing abnormal autoantibodies and finally trigger irAEs [105, 106].

##### Abnormal TPOAb in thyroid dysfunction

Endocrine toxicity is also a common irAE in immunotherapy. Up to 40% of patients have endocrine toxicity [107, 108], among which thyroid toxicity is the most common [109], mostly occurring 1–3 months after ICI treatment [109, 110]. Compared with CTLA-4 checkpoint inhibitors, PD-1 inhibitors are more prone to hypothyroidism, while compared with PD-L1 inhibitors, they might lead to the opposite conclusion: hyperthyroidism [111]. Stefano Gay et al. affirmed an interesting association between widespread thyroid hypoechogenicity, decreased thyroid volume, and elevated TPOAb after ICI treatment and thyroid dysfunction in patients with NSCLC or malignant pleural mesothelioma treated with ICIs [33]. This is consistent with the conclusion mentioned above that patients with positive anti-thyroid antibodies are more likely to develop thyroid dysfunction [32]. The decreased thyroid volume might be due to abnormal thyroid function. Additionally, hypoechoic grade and TPOAb elevation have been demonstrated to be a marker of the lymphocytic infiltration of thyroid parenchyma [112, 113]. This also corresponds with the hypothesis that ICIs enhance T cell activity against antigens present in healthy tissues [7] and increase pre-existing autoantibody levels [105, 106].

##### Anti-GNAL and anti-ITM2B in Hypophysitis

Hypophysitis is an inflammatory disease of the pituitary gland. The main pathological change is that the pituitary gland is infiltrated by immune cells, resulting in the destruction of endocrine cells and the expansion or atrophy of the pituitary gland [114–116]. Pituitary inflammation was more likely to occur with CTLA-4 inhibitors than with PD-1/PD-L1 inhibitors [117]. Similar pathological changes were found in immune-related hypophysitis and autoimmune hypophysitis [118]. Salahaldin A. Tahir et al. took this as inspiration and identified that elevated levels of anti-GNAL and anti-ITM2B autoantibodies (a median of 1.7-fold and 2.5-fold increase for GNAL and ITM2B, respectively, compared to pretreatment samples) might contribute to immune-related hypophysitis by taking advantage of the large-scale screening of autoantibodies in plasma [31]. GNAL and ITM2B, as target proteins expressed in the pituitary gland epithelium, are established as key signal molecules in the normal secretion of various pituitary hormones, such as TRH [119, 120] and ACTH [121, 122], and the increase in anti-GNAL and anti-ITM2B autoantibodies is a manifestation of the destruction of pituitary function. Currently, the prevailing possible mechanism is the qualitative difference in autoreactive effector T cells between anti-PD-1/PD-L1 and anti-CTLA-4 treatment [123]. Anti-CTLA-4 could generate new pituitary reactive effector T cells by activating recent thymic emigrants to diversify the immune response [123], while anti-PD-1/PD-L1 is more likely to take effect in making existing pituitary reactive effector T cells more active by releasing pre-existing effector T cells [124]. Another hypothesis is that the pituitary endocrine cells themselves might express CTLA-4, making the hypophysis a direct target for anti-CTLA-4 antibodies and causing hypophysis destruction [125]. Furthermore, these results enlighten us that in addition to increased autoantibody levels, disorders of pituitary hormone levels regulated by GNAL and ITM2B, such as TRH and ACTH, as another sign of impaired pituitary function, might also become a promising new research direction to predict pituitary inflammation, but it is expected to be supported by theoretical studies in the future.

#### Dermatologic toxicity

##### HLA allele

Derma-related adverse events mainly present as vitiligo [126, 127], rash and erythema [96], and cases of Stevens-Johnson syndrome and toxic epidermal necrosis are rarely reported [128]. As mentioned above, patients with preexisting abnormal RF are more likely to have derma-related adverse events after ICI treatment [9]. A retrospective study found that HLA-drb1 *11:01, a common genetic variant of HLA, was observably related to itching [30]. It is worth noting that dermatological irAEs have been demonstrated to imply universal activation of the immune system [129]. One proposed hypothesis is that there are potential shared antigens between tumor cells and normal tissue, such as skin, that could be recognized by antigen-specific T-cells, enhancing T cell recognition of skin antigens [96], and the HLA gene plays a vital role in antigen expression and immune tolerance [83], mediating a variety of autoimmune diseases [130].

##### IL-17

Daniel Johnson et al. reported a case of psoriasiform dermatologic toxicity in a melanoma patient that was induced by a PD-1 inhibitor and subsided after treatment with systemic IL-17A blockade [34]. Th17 cells can induce the transcription of IL-17 cytokines, which play a momentous role in a set of autoimmune diseases [131]. However, the blocking of CTLA-4 and PD-1 could increase the expression of Th17 cells in peripheral blood [132, 133]. Based on the crucial role of the Th17/IL17A axis in dermatologic diseases, such as psoriasis, it is hypothesized that ICIs enhance the Th17-mediated immune response in susceptible patients, thus causing some immune-related toxicities.

## Discussion

Many biomarkers have been claimed to predict irAEs, but unfortunately, none is perfect. Currently, research on irAE biomarkers is still in its infancy, with little progress and some deficiencies. First, there is a clinical challenge that some biomarkers are not routine tests in clinical diagnosis and treatment, and the relatively high cost will severely limit their clinical application. Therefore, it is important to develop simple, measurable and accurate biomarkers for personalized patient management. Additionally, by reviewing the biomarkers that predicted irAEs, we found that little is known about the prediction of some fatal irAEs, such as cardiotoxicity, neurotoxicity, and hepatotoxicity. The reasons might be as follows: on the one hand, the incidence of fatal irAEs is low, which leads to a small sample size; on the other hand, the patients cannot cooperate well due to the high mortality and side effects of fatal irAEs. Therefore, these valuable clinical cases are of vital importance and need to be collected and analyzed carefully to solve this problem. Moreover, since the mechanisms of irAEs induced by ICIs are very complex and involve many factors, the discovery of new potential biomarkers will provide insights into the mechanisms of the early prediction and resolution of irAEs in clinical practice. Another key issue is the unicity of irAE treatment. Currently, corticosteroids remain the basis of treatment, and in severe cases, biological immunoregulatory drugs are needed [134]. However, high-dose corticosteroid therapy may bring long-term and potential adverse events to patients, leading to femoral head necrosis, electrolyte disturbance, aggravation of cardiovascular and cerebrovascular diseases, etc. The lack of individualized treatment might delay the effective control of irAEs, and thorough mechanistic research and translational research in the future might provide opportunities for new treatment methods. The well-researched pathogenesis of irAEs might provide more targeted interventions to reduce the systemic adverse reactions caused by hormone shock therapy. At present, some clinical studies attempt to combine some modulators of the immune microenvironment with immunotherapy to further improve the efficacy, such as the Keynote-037 study [135] and Keynote-252 study [136]. Therefore, the development of drugs to regulate the occurrence of irAEs may be a future research direction that is expected to provide new strategies for the precise treatment of irAEs.

Many studies have shown that patients suffering from irAEs are more likely to achieve a longer survival benefit, which is a signal of lasting efficacy [70]. Therefore, could this correlation be used as evidence to support the deduction that biomarkers related to efficacy could also serve to predict the occurrence of irAEs? Indeed, there are some biomarkers that could predict not only the outcome of receiving ICI therapy but also the occurrence of irAEs. For example, the increase in IL-6 levels could also be used as a nonspecific biomarker to predict irAEs while being associated with poor treatment response [43]. The increased level of sCTLA-4 was applied to predict not only the good antitumor effect of anti-CTLA-4 but also the higher possibility of irAEs [26]. However, there are still some biomarkers, such as soluble PD-L1 (sPD-L1), that are only used to predict the efficacy of immunotherapy in current studies. Increased sPD-L1 levels are significantly linked with better treatment response in patients treated with anti-PD-1 therapy for NSCLC [137]. Conversely, in patients with multiple solid tumors taking PD-1/PD-L1, lower sPD-L1 levels were revealed to be associated with better treatment responses and survival outcomes [138, 139]. Therefore, this paradox still needs to be addressed by a larger cohort, but no relevant studies have yet shown a link between sPD-L1 and irAEs; the connection between them is expected to be revealed in the future. Future research could focus on the discovery of new and convenient markers for predicting the occurrence of irAEs. Moreover, it might also be a good direction to explore biomarkers that can simultaneously predict the reaction efficacy and the occurrence of irAEs, which has crucial clinical value.

Admittedly, there are some limitations to this review. First, since most of the studies we included were retrospective studies, they may lead to certain selection bias and confounding bias. However, this limitation is mostly due to the lack of thorough studies and a series of randomized controlled trials to provide evidence. More prospective studies and further molecular mechanism exploration are expected to make up for this deficiency. In addition, some original literatures are excluded due to lack of sufficient credible evidence and complete experimental protocol and results presentation, which to some extent, may lead to incomplete retrieval of literature. Therefore, in order to understand the biomarkers associated with irAEs as comprehensively as possible, we strictly followed the retrieval strategy to review the literatures.

## Conclusion

In conclusion, with the advent of the era of combined immunotherapy, the application scope and treatment population of immunotherapy are expanding. IrAEs, which affect multiple organs, have become a major obstacle to the application prospect of ICIs. The cautious management of irAEs, especially early detection and treatment, can facilitate achieving the maximum clinical benefit from immunotherapy. Extensive interdisciplinary cooperation to provide the best treatment options and the comprehensive consideration of multiple biomarkers to predict irAEs are also keys to the successful delivery of personalized treatment strategies. Additionally, irAEs can be used to screen and exclude people unsuitable for immunotherapy, which may provide another strategy for precise immunotherapy.

## Supplementary Information

**Additional file 1 **: **Supplementary Figure 1.** The quality evaluation of studies related to non-specific biomarkers.

**Additional file 2 **: **Supplementary Figure 2.** The quality evaluation of studies related to organ-specific biomarkers.

## Data Availability

No Applicable.

## Abbreviations

ICI: Immune checkpoint inhibitor
NSCLC: Non-small cell lung cancer
irAEs: Immune-related adverse events
CTLA-4: Cytotoxic T-lymphocyte antigen-4
IPI: Ipilimumab
PD-1: Programmed cell death protein 1
PD-L1: Programmed cell death ligand-1
CRP: C-Reactive protein
ULN: Upper limit of normal
Treg cell: T regulatory cells
IL-6: Interleukin − 6
Th: Helper T cells
CRS: Cytokine release syndrome
CAR -T cells: Chimeric antigen receptor T- cells
ALC: Absolute lymphocyte counts
AEC: Absolute eosinophil counts
NLR: Neutrophil-lymphocytes ratio
PLR: Platelets-lymphocytes ratio
ELR: Eosinophil-lymphocytes ratio
IBD: Inflammatory bowel disease
CSF: Colony-stimulating factor
TMB: Tumor mutation burden
sCTLA-4: Soluble CTLA-4
OS: Overall survival
GI: Gastrointestinal irAEs
HLA: Human leukocyte antigen
RF: Rheumatoid factor
